# COVID-19 and the production of knowledge regarding recommendations during pregnancy: a scoping review^*^

**DOI:** 10.1590/1518-8345.4523.3348

**Published:** 2020-06-26

**Authors:** Victor Hugo Alves Mascarenhas, Adriana Caroci-Becker, Kelly Cristina Máxima Pereira Venâncio, Nayara Girardi Baraldi, Adelaide Caroci Durkin, Maria Luiza Gonzalez Riesco

**Affiliations:** 1 Universidade de São Paulo, Escola de Enfermagem, São Paulo, SP, Brazil.; 2 Scholarship holder at the Conselho Nacional de Desenvolvimento Científico e Tecnológico (CNPq), Brazil.; 3 Universidade de São Paulo, Escola de Artes, Ciências e Humanidades, São Paulo, SP, Brazil.; 4 Kettering College, Nursing, Kettering, OH, USA.

**Keywords:** Obstetric Nursing, Pregnancy, Coronavirus Infections, Obstetrics, Prenatal Care, Infectious Disease Transmission, Vertical, Enfermagem Obstétrica, Gravidez, Infecções por Coronavírus, Obstetrícia, Cuidado Pré-Natal, Transmissão Vertical de Doença Infecciosa, Enfermería Obstétrica, Embarazo, Infecciones por Coronavirus, Obstetricia, Atención Prenatal, Transmisión Vertical de Enfermedad Infecciosa

## Abstract

**Objective:**

to map the production of knowledge regarding recommendations for providing care to pregnant women dealing with the novel coronavirus.

**Method:**

scoping review, using a broadened strategy to search databases and repositories, as well as the reference lists in the sources used. Data were collected and analyzed by two independent reviewers. Data were analyzed and synthesized in the form of a narrative.

**Results:**

the final sample was composed of 24 records, the content of which was synthesized in these conceptual categories: clinical manifestations, diagnosis, treatment, working pregnant women, vaccine development, complications, prenatal care, vertical transmission, and placental transmissibility. It is recommended to confirm pregnancy and disease early on, to use technological resources for screening and providing guidance and support to pregnant women.

**Conclusion:**

recommendations emphasize isolation, proper rest, sleep, nutrition, hydration, medications, and in the more severe cases, oxygen support, monitoring of vital signs, emotional support, and multiprofessional and individualized care. Medications should be used with caution due to a lack of evidence. Future research is needed to analyze the impact of the infection at the beginning of pregnancy and the psychological aspects of pregnant women infected with the virus.

## Abstract (pt)

**Objetivo:**

mapear a produção de conhecimento sobre as recomendações para a assistência à gestante no enfrentamento do novo Coronavírus.

**Método:**

revisão de escopo, com estratégia de busca aplicada aos bancos de dados e repositórios, bem como nas listas de referências das fontes utilizadas. A extração dos dados e a análise do material recuperado foram feitas por dois revisores independentes e os dados foram analisados e sintetizados em forma de narrativa.

**Resultados:**

a amostra final foi composta por 24 registros, que tiveram os conteúdos sintetizados nas categorias conceituais: manifestações clínicas; diagnóstico, tratamento; gestante na atividade laboral; desenvolvimento de vacinas; complicações; pré-natal; transmissão vertical e transmissibilidade via placentária. Recomendam-se a importância da confirmação precoce da gravidez e da doença, a utilização de recursos tecnológicos para triagem, a orientação e o suporte à gestante.

**Conclusão:**

dentre as orientações, tem-se que o foco da assistência deve incluir isolamento, repouso, sono, nutrição, hidratação, medicamentos e, em casos mais graves, suporte de oxigênio, monitorização dos sinais vitais, atenção emocional e cuidado multiprofissional e individualizado. Medicamentos devem ser utilizados com cautela, pois faltam evidências. Estudos futuros são necessários para analisar o impacto da infecção no início da gestação e os aspectos psicológicos de gestantes infectadas.

## Abstract (es)

**Objetivo:**

mapear la producción de conocimientos sobre las recomendaciones para la atención a las embarazadas en el enfrentamiento del nuevo coronavirus.

**Método:**

revisión del alcance, con estrategia de búsqueda aplicada a las bases de datos y los repositorios, así como en las listas de referencia de las fuentes utilizadas. La extracción de los datos y el análisis del material recuperado fueron realizados por dos revisores independientes y los datos se analizaron y sintetizaron en forma de narrativa.

**Resultados:**

la muestra final estaba compuesta por 24 registros y los contenidos fueron sintetizados en las categorías conceptuales: manifestaciones clínicas, diagnóstico, tratamiento, embarazada en el trabajo, desarrollo de vacunas, complicaciones, prenatal, transmisión vertical y transmisibilidad a través de la placenta. Se recomienda la importancia de la confirmación temprana del embarazo y de la enfermedad, el uso de recursos tecnológicos para la clasificación, la orientación y el soporte a la embarazada.

**Conclusión:**

entre las directrices, se señala que el foco de la atención debe incluir aislamiento, descanso, sueño, nutrición, hidratación, medicamentos y, en casos más graves, soporte de oxígeno, monitorización de los signos vitales, atención emocional y atención multiprofesional e individualizada. Medicamentos deben usarse con precaución, porque faltan evidencias. Estudios futuros son necesarios para analizar el impacto de la infección al inicio de la gestación y los aspectos psicológicos de embarazadas infectadas.

## Introduction

On December 31^st^, 2019 China reported to the World Health Organization pneumonia cases of unknown etiology in the city of Wuhan, province of Hubei. On January 9^th^, 2020 the coronavirus, scientifically known as Severe Acute Respiratory Syndrome-Coronavirus (SARS-CoV-2), was identified as the most recent microorganism causing the human infection called COVID-19. Since then, this virus has crossed Chinese borders and caused a devastating pandemic, challenging health services and the society and leading to high levels of mortality that varies according to each country’s epidemiological and social characteristics^(1-4)^.

The dissemination of this disease led the WHO to declare on January 30^th^, 2020 a “Public Health Emergency of International Importance”, which requires actions to prevent its transmission and decrease the occurrence of new infections. Recommendations include the early detection of the disease, social isolation for the entire community, the reporting of cases, and to investigate and properly manage cases^(5-6)^.

It is known that the main routes of SARS-COV-2 transmission are droplets of secretions from the respiratory tract of symptomatic or asymptomatic individuals who carry the virus, and contaminated objects. There is evidence that this pathogen is transmitted through feces^(4)^. There has been much effort to contain the contamination, considering that individuals with the SARS-COV-2 virus may be asymptomatic. When symptomatic, people tend to experience: fever, running nose, nasal congestion, dyspnea, malaise, loss of taste, and even more severe symptoms such as SARS. Complications are most common, and even lethal, among elderly individuals, immunosuppressed individuals, women during pregnancy and post-partum, and those with comorbidities^(3,7)^.

There is a gap in the knowledge concerning the implications of SARS-COV-2 during pregnancy. Initially, the number of pregnant women infected was proportionally smaller than the population in general, however, when infected, these women became more vulnerable to the more severe manifestations of the disease^(8-10)^.

In this sense, in March 2020, the Brazilian Ministry of Health included pregnant women as a risk group for COVID-19 based on physiological changes that take place during pregnancy, which tend to aggravate infectious conditions due to the low tolerance to hypoxia observed in this population^(6,11)^. The decision to include pregnant women as risk group took into account prior knowledge regarding other viruses and even respiratory infectious caused by the H1N1 virus among pregnant women, which resulted in high rates of complications and mortality^(11)^.

Despite the sensible concern of international and national health agencies^(4,11)^, there is little scientific evidence on the novel coronavirus and even less evidence regarding the management of pregnant women testing positive for SARS-CoV-2 or suspected to have the infection. Therefore, given this scenario, the objective of this review is to map the production of knowledge regarding recommendations on care provided to pregnant women dealing with the novel coronavirus.

## Method

This is a scoping review, defined as a way to map the main concepts that ground a field of research. Because of the emergency of this topic and the low availability of scientific evidence on the topic, the choice for this methodology is justified because it contemplates all kinds of scientific literature, going beyond issues concerning the effectiveness of an intervention or experience with treatments or care. Five stages were followed in this study, as listed by Arksey and O’Malley, namely: identification of the research question, identification of relevant studies, selection of studies, mapping of information, and grouping, summary and report of results^(12-13)^.

The question guiding this review was: “What is the production of knowledge regarding recommendations for providing care to pregnant women dealing with the novel coronavirus?”. The studies included in this scoping review were selected based on the mnemonic strategy PCC (Population, Concept, and Context), as recommended by the Joanna Briggs Institute (JBI) protocol. The population in this review was pregnant women, the concept of interest was COVID-19 pandemic and SARS-CoV-2 virus, and the context analyzed was pregnancy.

The search and selection of papers was performed in the databases appropriate for the topic under study: Medical Literature Analysis and Retrieval System Online - MEDLINE^®^ (access via PubMed), Scopus, Cumulative Index to Nursing and Allied Health Literature (CINAHL), Web of Science (WoS), and Latin American and Caribbean Center on Health Sciences Information (LILACS), through three different stages: 1) controlled descriptors appropriate for the databases were used in the first search (Medical Subject Headings - MeSH, CINAHL Headings and Health Sciences Descriptors - DeCS); 2) in the second stage, non-controlled descriptors were used in all databases and repositories to broaden the search and use terms that were specific to the current topic; 3) the third stage consisted of identifying and selecting reference lists on the sources used. Note that it was not possible to include gray literature due to the currency of the topic researched.

**Figure 1:**
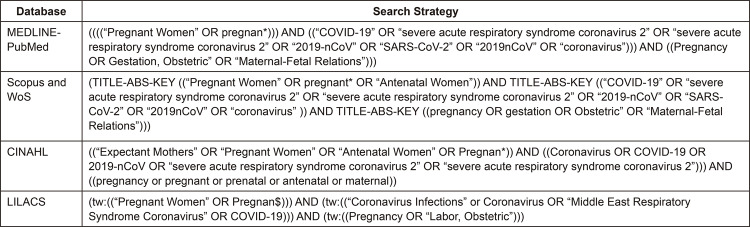
– Search strategies used in the databases. São Paulo, Brazil, 2020

The search strategy used in the different databases is described in Figure 1:

Inclusion criteria were studies with a wide range of methodologies (primary studies, literature reviews, editorials, and guidelines), written in English, Spanish or Portuguese, published up to March 2020, specifically addressing the novel coronavirus that causes COVID-19 in the context of pregnancy and outcomes in maternal-child health. Papers that did not meet the study’s objectives or did not provide pertinent information were excluded.

The Endnote Web software available online was used to properly store and organize the references obtained in the search. It allows for more than one researcher to automatically access the references, which is an important resource during the study selection process. Two independent reviewers accessed the same search results and verified the relevance of the studies identified. Disagreements regarding the inclusion of papers were resolved through a discussion among peers or the assessment of a third reviewer.

The methodological quality of the primary studies was not assessed, as this aspect is not taken into account in scoping reviews, however, data were collected using a form recommended by JBI, which is intended to facilitate the synthesis of information and quality of recommendations^(14)^. Data were collected using an instrument that was adapted from this form to map information. This instrument addresses: publication information (year, authors, country of origin); study’s objectives; methodological characteristics (characteristics of the study population); main results (outcome measures and main findings or contributions); context (care setting and relevant cultural and social factors)^(12,13)^. The results collected were presented in tables and discussed in the form of a narrative, based on the classification of conceptual categories.

The PRISMA checklist was adopted to ensure the quality of this study as it ensures the parts composing this review are appropriate^(15)^.

## Results

**Figure 2:**
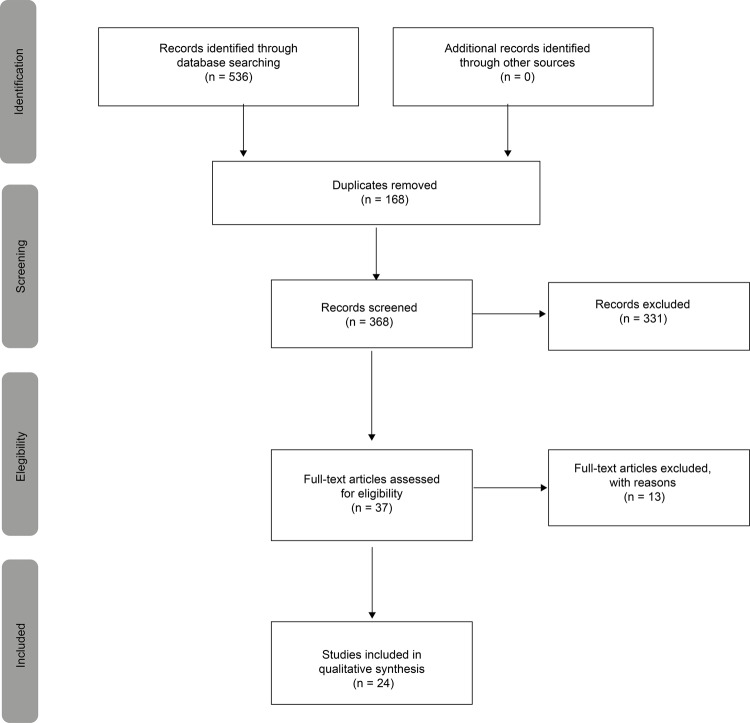
– Flow diagram of the review study selection, PRISMA-ScR. São Paulo, Brazil, 2020

Regarding the selection and inclusion of papers, the specific PRISMA extension for scoping reviews (PRISMA-ScR) was used, which is ideal to describe in detail the decision process of research considering the method used^(16)^. As shown in Figure 2, a total of 536 studies were potentially eligible (MEDLINE/PubMed=188; Scopus=262; WoS=55; CINAHL=29; LILACS=2). Of these, 168 studies were excluded because they were duplicates, as detected by Endnote Web. Thus, 368 studies were selected for the stage of reading titles and abstracts, from which 37 articles were eligible. Thirteen of these were excluded either because their full texts were not available or were incongruent with this study’s objectives. Thus, the final sample was composed of 24 articles, the full texts of which were read and analyzed by two researchers and authors of this study.

Most of the papers were written by Chinese researchers (n=14), followed by papers published in England (n=4), United States of America (USA) (n=4), and Singapore (n=2).

**Figure 3:**
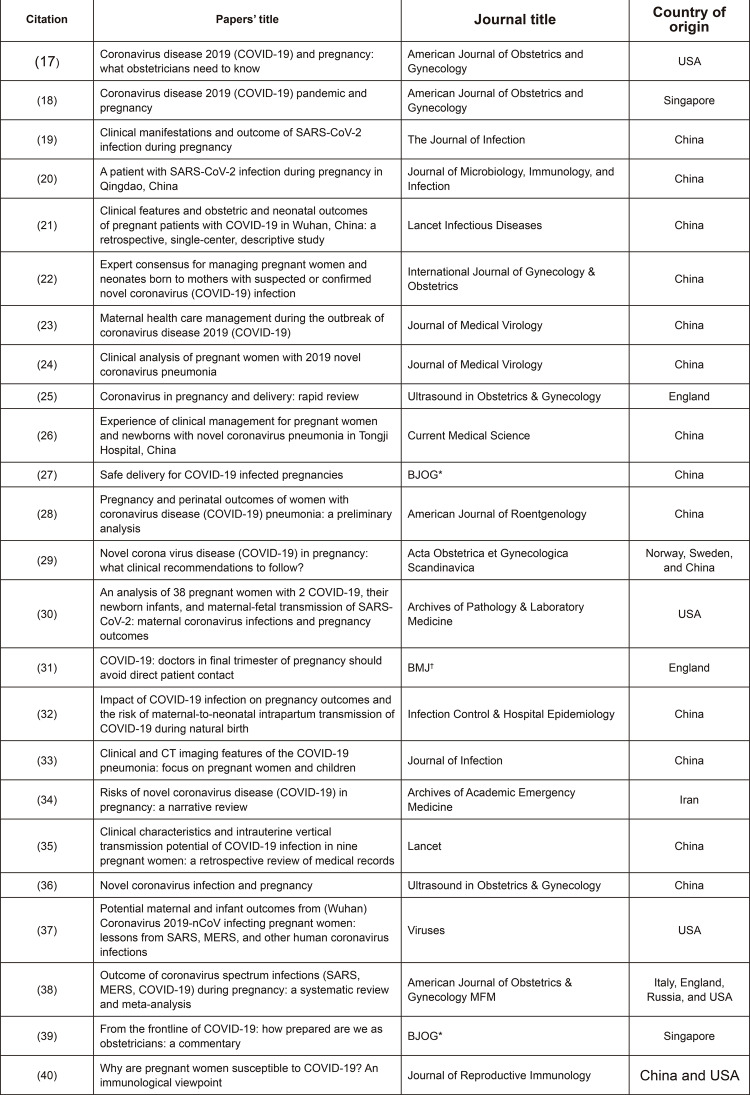
– Studies included by the scoping review according to the title, periodical, and country of origin. São Paulo, SP, Brazil, 2020 ^*BJOG =^ British Journal of Obstetrics and Gynaecology; ^†^BMJ = British Medical Journal

All the studies were published in 2020, were written in English and published in different periodicals, not limited to those specifically from the fields of obstetrics and gynecology, but also included the fields of epidemiology, infectious diseases, microbiology, immunology, pathology, radiology, and pediatrics. The studies’ specific characteristics are presented in detail in Figure 3.

Regarding the papers’ methodological designs, there were empirical studies (n=12) and theoretical studies (n=12). Eight were retrospective descriptive studies, six were reviews, five were opinion papers, three were case studies, and two were experience reports.

**Figure 4:**
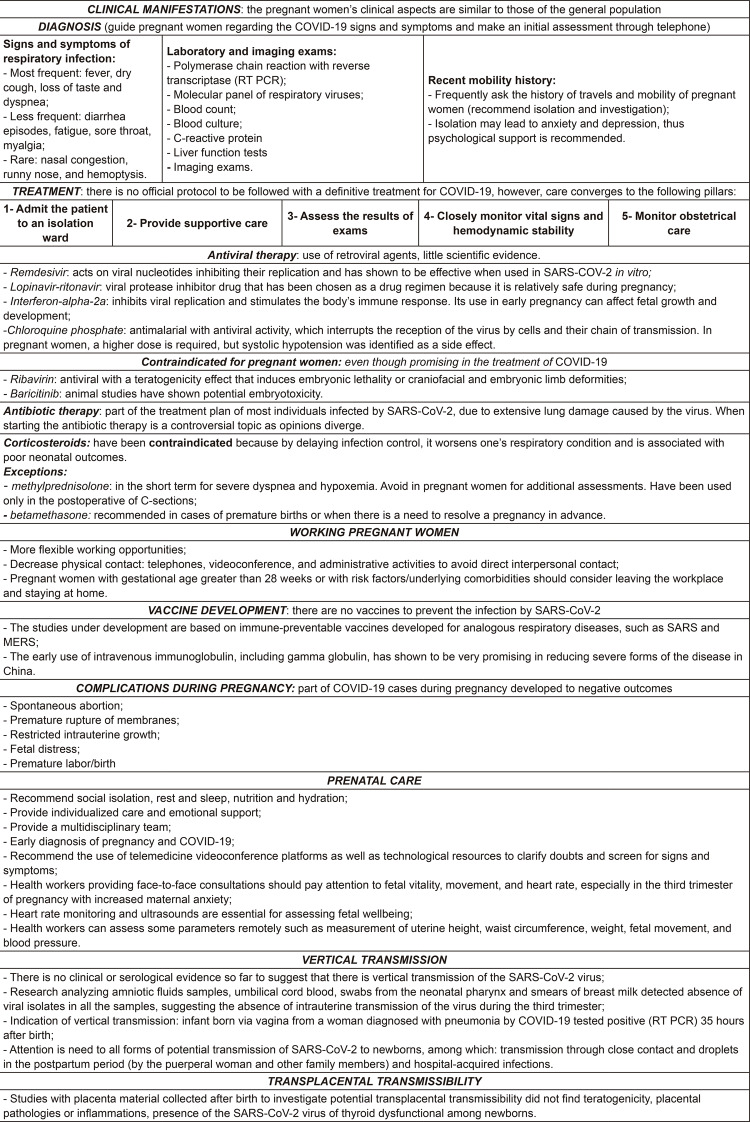
– Main recommendations for providing care to pregnant women dealing with the novel coronavirus. São Paulo, Brazil, 2020

The following conceptual categories emerged from the results obtained from the studies analyzed: *clinical manifestations, diagnosis, treatment, working pregnant women, vaccine development, pregnancy complications, prenatal care, vertical transmission, and transplacental transmissibility.* Figure 4 is intended to make recommendations objective and facilitate access to the main information.

## Discussion

This scoping review made it possible to map the body of knowledge concerning recommendations to the care provided to pregnant women dealing with the novel coronavirus. The Brazilian Ministry of Health classified pregnant women as a risk group and provided recommendations as they tend to present poor outcomes when contaminated^(8-9,11)^.

The pandemic caused by the novel coronavirus remains very serious, highly contagious, and has affected the world population beyond risk groups. Note the importance of sensitizing and making individuals aware of its severity to reinforce preventive measures to decrease and control this infection^(4,41-42)^.

Most of the studies were conducted in China because it is where the novel coronavirus originated. Additionally, even though most studies present few clinical cases, attention should be paid to these studies as they report evidence that is currently available and it is extremely relevant to identify the main clinical manifestations dealing with this disease during pregnancy.

No differences were found between the clinical aspects manifested by pregnant women infected with COVID-19 and those of non-pregnant individuals^(17-20,28,34-36,38)^. The main symptoms reported were: fever^(18-21,28-29,32,34,36,39)^, dry cough^(18,20,28-29,31-32,34,36,38-39)^, and dyspnea^(18-19,21,29,32,36)^. Nevertheless, a review addressing COVID-19 during pregnancy reports other signs and symptoms, which even if at a lower frequency, may occur and should be taken into account to reach an early diagnosis^(29)^.

In terms of diagnosis, there is a concern with early detection of the disease. Thus, pregnant women should be recommended to learn about the specific signs of COVID-19 in order to decrease their exposure to health services. An initial assessment performed online is recommended to determine whether a face-to-face consultation is necessary^(17)^. Note, however, that a retrospective study^(33)^ compared the clinical aspects of pregnant and non-pregnant women and verified that symptoms are atypical among pregnant women, which would probably hinder the disease’s early detection in this group.

For detection in the presence of specific symptoms, studies suggest: laboratory exams^(6,36,29)^ and complementary imaging exams^(18,28-29,33,35-36)^. Comparative studies indicate that CT scans are more sensitive than RT-PCR, as well as more precise and time-efficient, presenting a lower number of false-negatives. The clinical findings of imaging exams of pregnant women are similar to those of non-pregnant patients^(28-29,33,35)^. Despite its various advantages, the routine use of CT scans should be avoided due to the risk of exposure to radiation. Note that none of the radiological exams replaces the molecular confirmation of COVID-19^(18)^.

As for the treatment of positive pregnant women, there is not a consensual and official protocol so far. Hence, the medications and conducts are subject to cultural and health care contexts, though the main axes of care are based on isolating pregnant women, classifying them according to risks and needs determined by their clinical condition; recommending proper sleep and rest; promoting appropriate nutrition; providing supplementary oxygen support, if needed; and monitoring the intake of fluids and electrolytes. Vital signs and oxygen saturation levels should be closely monitored, as well as the frequency of the fetus’ heart rate to observe the progression of the pregnancy, planning individualized delivery, and having a multiprofessional team to provide care^(17-18,22,29,36,39)^.

Amid this pandemic, it is also important to keep in mind that health workers should ensure women the right to humanized care to be provided during pregnancy, delivery, and puerperium, as well as to the child the right to have a safe birth, and healthy development and growth. In Brazil, these rights are ensured by the *Rede de Atenção à Saúde Materna e Infantil* [Maternal and Child Health Care Network] known as *Rede Cegonha* [Stork Network] and instituted through Ordinance No. 1459/2011^(43)^.

The studies also highlight that this time requires pregnant women have more flexible work opportunities, being able to leave from work when gestational age is greater than 28 weeks or when there are risk factors or underlying comorbidities^(31,39-40)^. These precautions are needed considering that being infected by COVID-19 during pregnancy tends to result in negative outcomes, such as spontaneous abortion, premature rupture of membranes, restricted intrauterine growth, fetal distress, and premature labor and birth^(18-19,24,27-29,32,34,36,38-40)^.

Thus, in this context, prenatal care is essential throughout the pregnancy, especially during the third trimester when the final stages of development take place and maternal anxiety is at its highest, a period that requires a larger number of prenatal inspections. Therefore, monitoring heart rate and ultrasound are essential to assess fetal wellbeing, and especially among women infected with the novel coronavirus^(18,23,26)^.

Note that so far, there is no clinical or serological evidence suggesting the possibility of vertical transmission of the SARS-CoV-2 virus^(6,17-19,25,27-28,30,32,34,38-40)^ in amniotic fluid samples^(17-18,25,30,34)^, umbilical cord blood^(17-18,25,30,32,34,38-40)^, newborn pharyngeal swabs^(17-18,25,30,32,34,39)^, or milk breast smears^(18,25,30,32,34-35,39)^. An absence of isolated viral was verified in all the samples, suggesting there is no intrauterine transmission of the virus during the third trimester. All the studies, however, are retrospective studies addressing small samples, characteristics that decrease the power of generalization. Studies using placentas also identified no teratogenicity, placental pathologies or inflammations, the presence of the SARS-CoV-2 virus, or thyroid dysfunction in newborns^(18,25,29,34,36-37)^.

This scoping review’s limitations include the fact that the recent beginning of the pandemic and intense flow of information prevents the supply of stable recommendations. As most studies are retrospective studies and opinion papers, there is the risk of biased information. Additionally, the option to restrict studies written in one of three languages also limited the number of studies, as potentially eligible papers originated in China were written in the native language.

## Conclusion

Pregnant women represent a group with particularities, especially linked to physiological and immunological changes. Additionally, the need to protect a fetus represents greater responsibility when providing care.

This review mapped all information available so far regarding the care provided to pregnant women during the COVID-19 pandemic. There is much uncertainty regarding the virus-specific characteristics, however, the following is recommended to promote quality care to the maternal-fetal pair: contain as much as possible the advancement of the virus with isolation and contact precautions; care for respiratory infections; assess risks and benefits constantly; confirm the disease and pregnancy the earliest as possible; use technological resources for screening; provide oxygen support if needed; recommend proper rest, sleep, nutrition, and hydration; use medications when indicated and contraindicated medications that may present teratogenic or toxic effects to the fetus; monitor vital signs; provide individualized obstetrical care with a multiprofessional approach.

The information presented is not absolute and may change when new scientific discoveries are reported. The results of the studies included in this review support future studies to investigate the impact of the infection at the beginning of pregnancy (during the first and second trimesters), the psychological aspects of infected pregnant women, and analyses of medications specific for pregnancy. The gaps that remain are expected to motivate the development of further research with greater methodological rigor, to produce reliable scientific evidence concerning obstetrical care provided in the context of the COVID-19.
